# Utilization of routine health information and associated factors among health workers in Hadiya Zone, Southern Ethiopia

**DOI:** 10.1371/journal.pone.0233092

**Published:** 2020-05-21

**Authors:** Habtamu Wude, Mirkuzie Woldie, Dejene Melese, Tsegaye Lolaso, Bahailu Balcha

**Affiliations:** 1 East Bedawacho Health Office, Hadiya Zone, SNNPR, Shone, Ethiopia; 2 Department of Health Policy and Management, Institute of Health, Jimma University, Jimma, Ethiopia; 3 School of Public Health College of Health Science and Medicine, Wolaita Sodo University, Wolaita Sodo, Ethiopia; University of Ghana College of Health Sciences, GHANA

## Abstract

**Background:**

The utilization of routine health information is an essential factor of the structural capacity of health departments and public health performance depends on the effectiveness of information use for routine and programed decisions. Considerable research has been conducted in health data collection and ways to improve data quality, but little is known about utilization of routine health information among health workers in Ethiopia in general and in the study area in particular.

**Objectives:**

The aim of this study was to assess level of utilization of routine health information and associated factors among health workers in Hadiya zone, Southern Ethiopia, 2019.

**Methods and materials:**

Facility-based cross-sectional study design with both quantitative and qualitative data collection methods was employed at the Hadiya zone from March 10–25, 2019. A total of 480 health workers were included in the study and systematic random sampling was employed to select the health care workers in the study. The results were analyzed and presented in tables and graphs. Finally, the binary logistic regression was used to examine independent predictors.

**Result:**

Good level of utilization of routine health information was reported by 301 (62.7%, 95% CI: 58.5%-66.9%) of the health workers. Training [AOR = 8.12; 95% CI: (4.33–15.23)], supportive supervision [AOR = 2.34; 95% CI:(1.40–3.92)], good perceived culture of health information [AOR = 5.05; 95% CI: (2.99–8.50], having a standard set of indicators (AOR = 2.05 95%; CI: (1.23–3.41) and competence on routine health information tasks [AOR = 5.85; 95% CI: (3.41–10.02)] were independent predictors.

**Conclusion:**

Good level of utilization of routine health information was noted in less than two-third of the study participants. Further training, supportive supervision, perceived culture of health information, having standard set of indicators and competence on routine health information task were factors that improve routine health information utilization.

## Introduction

Health information is the processed data and knowledge that an individual or group use to support their decisions in the health sector [1]. It is fundamental for the overall health system which informs decision-making in each of the other five blocks of the system and improving managerial decisions by providing quality information for evidence-based health practices [2,3].

Routine health information is vital for operational, tactical, and strategic decision-making. Major problems in relation to health information which was identified by the World Health Organization(WHO) are inadequate use of existing information and evidences due to fragmentation and duplication of health information [4]. Thus, limited use remains the major concerns [5]. This leads to worldwide commitment to improving information utilization and to base decisions on reliable facts. In line with this the participants of the Global Summit on Measurement and Accountability for Health identified a five-point call to action with a set of targets for better data use in support of health-related sustainable development goals (SDG). One of this five-point call to action is ‘‘*by 2020*, *countries have health information flows that involve the use of data locally to improve services and programs*” [6].

Sub-Saharan African countries recognized and accepted Health Management Information System(HMIS) as a source of routine health information, however, health programs frequently fall short of its efficient use to inform decisions [7]. Further studies from this region showed that health workers usually spend 40% or more of their time in filling HMIS forms, but make little use of the information from this system [8]. Moreover, data seated in data banks, reports, or shelves were not processed and sufficiently utilized in program development, improvement, strategic planning and advocacy [8].

In Ethiopia, the generation and utilization of health information has been a priority since the period of the Health Sector Development Program four (HSDP-IV) [5,9]. Primarily the federal ministry of health manages Routine Health Management Information System and it has been a primary source of information for continuous monitoring of health services in the country. In response to the changes in health system organization, the Ethiopian federal ministry of health has undertaken revision of the routine health information system which is more comprehensive and focused on strengthening the standardization process through incorporating new initiatives [10].

Currently one of the four transformation agendas in the Health Sector Transformation Plan (HSTP) is “Information Revolution”. It involves a fundamental shift from old methods of information utilization to bring about a fundamental cultural and attitudinal change regarding practical use of information [10]. Despite these efforts, the utilization of routine health information at the local level is still a big challenge [11–13]. Health information is generated at each level of the health system and then submitted to each subsequent next level without adequate use for performance improvement at the point of generation [14]. Health workers in the health system are either not utilizing routine health information at all or not in a best way to drive planning, performance management, and the delivery of services. This creates hindrances to the efficiency and effectiveness of health care delivery [9,15]. The utilization of health information goes beyond the health sector in which health information is a base for different decision made in various sectors like communities, consumer groups, and non-governmental organizations [2,16]. It is vital for operational, tactical and strategic decision-making. However, poor data quality and limited use remain the major concerns [5]. Considerable research has been conducted in routine data collection and ways to improve data quality, but little is known about routine health information utilization and associated factors among health workers in Ethiopia in general and in the study area in particular. Further, there is no study conducted on routine information utilization in Southern Nation Nationalities People Region (SNNPR) and in Hadiya zone. Therefore, the aim of this study was to assess the level of utilization of routine health information and associated factors among health workers in Hadiya zone, Southern Ethiopia, 2019. The finding from this study will helps health workers to understand their routine health information utilization level and to design suitable intervention to improve evidence-based practice.

## Methods and materials

### Study design and setting

An institutional based cross-sectional study was conducted on 490 Health workers from March 10–25, 2019 in Hadiya Zone, southern Ethiopia. Hadiya zone is located in the Southern Nation Nationalities People Region, Ethiopia. Hosanna is the administrative center of Hadiya zone which is located 194 km far from regional city Hawassa to Southwest and 232 kilometers far from Addis Ababa in the Southern part of Ethiopia. This zone has one teaching hospital, three primary hospitals and 61 health centers. The zone currently has 2177 health workers from different disciplines working in hospitals and health centers.

### Sample size and sampling procedure

The sample size was determined by using both single and double population proportion formula. The following assumptions were used to calculate sample size for the first objective: 95% confidence level (1.96), 5% margin of error, and proportion (P) of the routine health information utilization rate 53.1% from similar study conducted in Eastern Ethiopia [17]. Accordingly, the sample size was calculated by using the formula: n = (z _(α/2)_)^2^p(1-p)/d^2^ and the sample size become, 383. Since the total population is less than 10,000, we used correction formula and considering the design effect of 1.5 due to multistage nature of the study; the sample size becomes 490.

The sample size for the second objective was determined using double population proportion formula with the assumptions of 95% CI, 5% margin of error, 80% power and unexposed to exposed ratio of 2.34 [18] and the sample size become 306. The final sample size becomes 490.

Multistage sampling was employed where in the primary stage of sampling, 6 districts were selected out of 12 districts in the Hadiya zone using the lottery method. This was based on the recommendation of the WHO [19]. Then the sample was allocated proportionally to each district based on size of health workers in each district. Then in the secondary stage of sampling, systematic random sampling was used to select health workers, based on the list that was available in the human resource department of each district health office.

### Data collection methods

Data was collected using a pretested structured questionnaire which was adapted from the performance of routine information system management (PRISM) framework tools. The questionnaire contains; socio-demographic part, level of routine health information use part, technical factors part, facility related factors part and individual behavioral factors part. Utilization of routine health information was assessed using ten items for which routine health information was utilized (use of routine health information for monitoring day to day health service activities, displaying updated information, developing weekly plan, service delivery improvement, drug procurement, resource mobilization, detecting the cause of health problem in the community, facilitating community mobilization, prediction of outbreaks, and disease prioritization) [18]. These were measured using items of 5-point Likert scale, 1 denoting never, 2 seldom, 3 sometimes, 4 often and 5 denoting almost always. Percentage score was calculated for each individual participant by summating scores on the routine health information utilization items and then dividing by 50 which was potential maximum score. Finally, the score was presented in percentages. For document review, checklist adapted from Federal ministry of Health HMIS guideline was used [20]. The main purpose of document review and observation was in order to verify the findings of quantitative method by reviewing records and resources related to routine health information. Data collectors reviewed records and resources at the office of health facility head and HMIS unit of the facility using the checklist. The document review was mainly focused on verification of the presence and content of performance review team meeting minutes related to routine health information, supervision feedbacks on RHIS and action plan to address gaps on utilization of routine health information. Observation at facility level focused on presence of key performance indicators charts, graphs and table displayed, presence of RHIS training manual and guide, and presence of RHIS supervisory checklist.

### Data management and analysis

The data were checked for completeness and consistency, and entered to Epidata software version 3.5 then exported to SPSS version 20 for further analysis. The internal consistency of the tools was checked (with Cronbach’s alpha = 0.95 for items measuring culture of health information, 0.72 for items measuring competence on routine health information tasks, 0.88 for items measuring motivation to use routine health information and 0.94 for items measuring the utilization of routine health information). Descriptive statistics was used to present the frequencies, proportion and summary statistics. Bivariate analysis was carried out to see the association of each independent variable with the routine health information utilization. Those variables with p-value less than 0.25 were included in multivariable logistic regression analysis. Multivariable logistic regression analysis was carried out to control possible confounders and identify factors independently associated with Routine information utilization. Finally, variables with p-value less than 0.05 in multivariable logistic regression analysis considered as independently significant association with Routine information utilization. The odds ratio was used to identify the strength of association with Routine information utilization. Prior to data collection ethical clearance was obtained from the institutional review board (IRB) in institute of health, Jimma University. Then informed written consent was obtained from the participants, after the necessary explanation about the purpose and benefits of the study and also the right on the decision of participating in the study.

## Results

### Socio-demographic characteristics of the respondents

Out of 490 health workers planned to participate, 480 participated resulting in a response rate of 98%. The mean age of respondents was 29.51 (SD ±4.20 years). The median age of respondents was 29 year. Around half 271(56.5%) of the respondents were male. Most 412(85.8%) of the participants worked in health centers while 68(14.2%) worked in hospital. With regard to work experience, the participants have the mean work experience of 4.5 (SD: ±3.23 years.) In terms of their position in the organization 401(83.5%) worked in expert position and 79(16.5%) worked in managerial positions. Regarding professional category, 200(41.7%) of participants were nurses, 75(15.6%) were public health officer, 72(15%) were laboratory professionals, 53(11%) were pharmacy professionals, 41(8.5%) were midwives and the remaining were others. They had a median monthly salary of 4085 ETB (Table 1).

**Table 1:** Socio-demographic characteristics of health workers working at public health facilities in Hadiya zone, Ethiopia, 2019, (n = 480).

Variables	Responses	Frequency	Percent
Age	20–24	58	12.1
	25–29	203	42.3
	≥ 30	219	45.6
Sex	Male	271	56.5
	Female	209	43.5
Professional categories	Nurse	200	41.7
	Public health officer	75	15.6
	Laboratory	72	15
	Pharmacy	53	11
	Midwife	41	8.5
	Others*	39	8.1
Educational level	Diploma	280	58.3
	Degree and above	200	41.7
Work experience	<5 years	297	61.9
	5–10 years	147	30.6
	>10 years	36	7.5
Type of institution	Hospital	68	14.2
	Health center	412	85.8
Department	OPD	145	30.2
	IPD	39	8.1
	Pharmacy	57	11.9
	Emergency	42	8.8
	MCH	112	23.3
	Laboratory	65	13.5
	Others	20	4.2
Position	Head position	79	16.5
	Expert position	401	83.5
Monthly income in ETB**	≤2400	22	4.6
	2401–2800	25	5.2
	>2800	433	90.2

*others were nutritionist, environmental health technicians and health informatics. ETB** = Ethiopian birr.

### Technical characteristics

Around two-third of the participants 311(64.8%) have not received training on routine health information system. More than half 277(57.7%) of the participants responded that they had standardized health indicators in their office. Of the total participants, 263(54.8%) reported that they have discussed the monthly performance progress using standard health indicators. The result from document review showed that 4/10 of the facilities conducted discussions witnessed by a performance review team meeting minutes on their performance and feedback from district as per the standard. More than two-third 335(69.8%) of the study participants reported that they had no data storage device in their working office. More than half 270(56.7%) reported that they have displayed health information (Table 2).

**Table 2:** Technical characteristics of health workers working at public health facilities of Hadiya zone, SNNRP, Ethiopia, 2019, (n = 480).

Variables	Categories	Frequency	Percentages (%)
Training on health information	Yes	169	35.2
	No	311	64.8
Time of training(n = 169)	Within 6 months	87	51.5
	6 months ago	82	48.5
Having standard health indicator	Yes	277	57.7
	No	203	42.3
Having displayed routine health information	Yes	270	56.3
	No	210	43.7
Discussion of performance using standard health indicators	Yes	263	54.8
	No	217	45.2
Change collected data in to information	Yes	285	59.4
	No	195	40.6
Regular report preparation	Yes	241	50.2
	No	239	49.8

### Facility related characteristics

Of the total participants 225(46.9%) have received supportive supervision for routine health information system and out of these about 185(82.2%) have been supervised either every month or quarterly in the year preceding the study period. Findings from observation also showed that only 3/10 of the facilities have health management information system supervisory checklists. Regarding the feedback, 224(46.7%) participants responded that they have received regular feedback on their report and out of these 100(45.2%) have received regular feedback for every report. Data from document review showed that just 4/10 of the facilities have feedbacks on routine health information use practice showing the strength and weakness of the system. Less than half 233(48.5%) of the participants have responded that they had regular evaluation for routine health information. Finally, out of total participants 265(55.2%) reported that they have got support from immediate supervisor on data management and information utilization (Table 3).

**Table 3:** Facility related characteristics at public health facilities of Hadiya zone, 2019, (n = 480).

Variables	Responses	Frequency	Percentages(%)
Supportive supervision	Yes	225	46.9
	No	255	53.1
Frequency of supervision(n = 225)	Every month	99	44
	Quarterly	86	38.2
	Semiannually	37	16.3
	Annually	3	1.3
Regular evaluation of RHI*	Yes	233	48.5
	No	247	51.5
Support from immediate supervisor	Yes	265	55.2
	No	215	44.8
Rating for support from immediate supervisor(n = 265)	Poor	29	10.9
	Fair	95	35.8
	Good	98	37
	Excellent	43	16.2
Availability of data management standards	Yes	290	60.4
	No	190	39.6
Regular meeting to improve RHI* utilization	Yes	272	56.7
	No	208	43.3
Regular feedbacks on RHI use practice	Yes	224	46.7
	No	256	53.3
Frequency of time participants analyze data	Never	107	22.3
	Occasionally	162	33.8

Regarding individual and behavioral characteristics, out of all participants 282(58.8%) had good perceived culture of health information; 253(52.7%) were motivated to use routine health information; 272(56.7%) have “high competence”.

### Routine health information utilization

In this study, good routine health information utilization was found among 301(62.7%) with (95% CI: 58.5%-66.9%) of the study participants. Furthermore, good RHI utilization was found among 45(66.2%) participants from hospital and 256(62.1%) participants from health centers.

### Factors associated with utilization of routine health information

The odds of utilization of routine health information were about eight times higher among trained individuals when compared with individuals who are not trained on routine health information [AOR = 8.12; 95% CI: (4.33–15.23)]. The odds of utilization of routine health information were about two times more among individuals who received supportive supervision on routine health information when compared with individuals who have not received supportive supervision [AOR = 2.34; 95% CI: (1.4–3.92)]. The odds of utilization of routine health information among study participants with high competence on routine health information tasks were about six times higher than their counter [AOR = 5.85; 95% CI: (3.41–10.02)]. The odds of utilization of routine health information were about two times higher among participants who have standard set indicators than their counterpart [AOR = 2.05; 95% CI: (1.23–3.41)] (Table 4).

**Table 4:** Factors associated with routine health information utilization among health workers in Hadiya zone, SNNRP, 2019, n = 480.

Variable	Response	Routine health information utilization		COR (95% CI)	AOR(95% CI)
		Good	Poor		
Position in the organization	Head position	58(73.4%)	21(26.6%)	1.79(1.05–3.07)	
	Expert position**	243(60.6%)	158(39.4%)	1	
Training on RHI	Yes	149(88.1%)	20(11.9%)	7.79(4.64–13.09)	8.12(4.33–15.23)*
	No**	152(48.9%)	159(51.1%)	1	1
Supportive supervision	Yes	173(76.8%)	52(23.2%)	3.30(2.22–4.90)	2.34(1.4–3.92)*
	No **	128(49.6%)	127(50.4%)	1	1
Having standard set of indicators	Yes	209(75.5%)	68(24.5)	3.70(2.51–5.47)	2.05(1.23–3.41)*
	No **	92(45.3%)	111(54.7)	1	1
Perceived culture of health information	Good	225(79%)	57(21%)	6.33(4.21–9.52)	5.05(2.99–8.50)*
	Poor**	76(38.4%)	122(61.6%)	1	1
Motivation to use RHI	Motivated	168(66.4%)	85(33.6%)	1.34(0.96–2.02)	
	Not motivated**	133(58.6%)	94(41.4%)	1	
Competence on RHI tasks	High	220(80.8%)	52(19.2%)	6.33(4.2–9.52)	5.85(3.41–10.02)*
	Low**	81(38.9%)	127(61.1%)	1	1
Data quality affect RHI use	Yes	184(66.67%)	92(33.33%)	1.48(1.02–2.16)	
	No**	117(57.35%)	87(42.6%)	1	

*Statistically significant at p<0.05.

**Reference category.

AOR = Adjusted odds ratio; COR = crude odds ratio; CI = confidence interval; RHI = Routine Health Information; CI = confidence interval; RHI = Routine Health Information.

## Discussion

This study had focused on the level of utilization of routine health information and determinants that affect it. Thus, the study revealed that 62.7% with (95% CI: 58.5%-66.9%) of the study participants had a good level of routine health information utilization. This finding was higher than the finding from studies conducted in different parts of Ethiopia, 38.4% in Northern Ethiopia [21], 53.1% in Eastern Ethiopia [17], 57.9% in Easter Wollega [9], 45.8% in Northwest Ethiopia [22], 54.2% in East Wollega [23]. This might be due to the fact that nowadays the government of Ethiopia in its HSTP plan has given a special focus to the utilization of health information in decision making and the improvement of health workers information using culture [10]. Similarly, the finding in this study is higher than earlier reports in Africa like 38% in Cote D’Ivoire [24], 53% in South Africa [25]. In contrast to the finding was lower than a study conducted in North Gondar zone in Amhara region which was 78.5% [18]. This may be due to the higher proportion of supervision (57.6% in the study conducted in North Gondar zone while it was 46.9% in the current study) and feedbacks (53.8% in study conducted in North Gondar zone while it was 46.7% in the current study). However a comparable level of utilization routine health information was reported from Tanzania (58%) [26].

Out of the variables which showed significant association with routine health information utilization, higher odds of routine health information utilization were found among health workers who had training on routine health information. Studies conducted in different parts of the world showed the fact that training is the most essential facilitator of the utilization of health information and could improve the potential of health workers to analyze and make evidence-based decision [26,27]. This finding is supported by a study conducted in Northern Ethiopia [28].

The odds of routine health information utilization among health workers who had supportive supervision were about two times higher than those of their counterparts. Supportive supervision has an important function in motivating health workers, identifying the gaps and improving health workers’ performance. Studies showed that supportive supervision fosters a collaborative approach to strengthening health worker performance [29]. The finding was supported by studies conducted in East Gojam, Northern Ethiopia [28] and East Wollega Zone, Oromia Regional State, Ethiopia [9] and other countries like Tanzania [26], rural of South Africa [27] and systematic review study in low and middle income countries [30].

The odds of routine health information utilization of health workers who had high competence on routine health information tasks were 5.85 times more when compared to health workers who had low competence on routine health information tasks. Competence to understand and interpret data stemmed from the routine health information system is one of the most promising facilitators for utilizing routine health information [25]. Accordingly, without processing data into information, it is not possible to utilize routine health information [30]. There is a need to improve information-use practices with better understanding of the health indicators and added skills on analysis and information use, and mentorship to all involved health workers [10]. This finding is supported by that of studies conducted in Western Amhara, Northern Ethiopia [28] and North Gondar zone, Ethiopia [18].

This study also showed that a good perceived culture of information enhances utilization of routine health information. The odds of routine health information utilization were about five times higher among participants who had good perceived culture of information-use compared to those who had a poor perceived culture of information-use. Studies show that inadequate use of information has been attributed to the lack of or an inadequate culture of information-use at facility levels [25]. This finding was supported by a study conducted in South Africa [27].

Finally, the odds of routine health information utilization among health workers who had standard health indicators in their offices were about two times higher than those who have no indicators. This might be due to the presence of data sources which offer utilization of information for evidence based decision making [31]. This is supported by study conducted in north Gondar [18].

## Conclusion

The moderate proportion of health workers showed a good level of routine health information utilization. Further training, supportive supervision, perceived good culture of health information, having a standard set of indicators and competence on RHI tasks were an independent predictor of routine health information utilization. This could call for regular supportive supervision, intensifying training and capacity building, mentoring on competence of routine health information tasks, improve a culture of health information and availing standard set of indicators.

## Supporting information

S1 Data (SAV)

## List of abbreviations

AOR: Adjusted Odds Ratio
CI: Confidence Interval
ETB: Ethiopian Birr
HMIS: Health Management Information System
HSDP: Health Sector Development Plan
HSTP: Health Sector Transformation Plan
IPD: Inpatient Department
IRB: Institutional Review Board
MCH: Maternal and Child Health
OPD: Outpatient Department
RHI: Routine Health Information
SDG: Sustainable Development Goals
SNNPR: South Nations Nationalities and Peoples Region
SPSS: Statistical Package for Social Sciences
WHO: World Health Organization
